# Effect of Pentacyclic Triterpenoids-Rich Callus Extract of *Chaenomeles japonica* (Thunb.) Lindl. ex Spach on Viability, Morphology, and Proliferation of Normal Human Skin Fibroblasts

**DOI:** 10.3390/molecules23113009

**Published:** 2018-11-17

**Authors:** Małgorzata Anna Kikowska, Małgorzata Chmielewska, Agata Włodarczyk, Elżbieta Studzińska-Sroka, Jerzy Żuchowski, Anna Stochmal, Małgorzata Kotwicka, Barbara Thiem

**Affiliations:** 1 Department of Pharmaceutical Botany and Plant Biotechnology, Poznan University of Medical Sciences, 61-861 Poznań, Poland; agata_nahorska@wp.pl (A.W.); bthiem@ump.edu.pl (B.T.); 2 Department of Cell Biology, Poznan University of Medical Sciences, 60-806 Poznań, Poland; mchmielewska@ump.edu.pl (M.C.); mkotwic@ump.edu.pl (M.K.); 3 Department of Pharmacognosy, Poznan University of Medical Sciences, 60-781 Poznań, Poland; elastudzinska@ump.edu.pl; 4 Department of Biochemistry and Crop Quality, Institute of Soil Science and Plant Cultivation, State Research Institute, 24-100 Puławy, Poland; jzuchowski@iung.pulawy.pl (J.Ż.); asf@iung.pulawy.pl (A.S.)

**Keywords:** *Chaenomeles japonica*, polyphenolics, pentacyclic triterpenes, antioxidant activity, in vitro cultures, skin fibroblasts

## Abstract

The effect of the well-characterized callus extract of *Chaenomeles japonica* on viability, morphology, and proliferation of normal human skin fibroblasts was investigated. The phytochemical analysis was performed using the ultra-high performance liquid chromatography method. The total phenolic, phenolic acid, and flavonoid contents were determined spectrophotometrically. The antioxidant activity was investigated using the DPPH (1,1-Diphenyl-1-picrylhydrazyl Radical Scavenging), FRAP (Ferric Reducing Antioxidant Power), and CUPRAC (CUPric Reducing Antioxidant Capacity) assays. The callus growth index during passages was high as well as the content of pentacyclic triterpenoids. The microscopic observations of the fibroblast viability, morphology and the evaluation of the proliferation ratio (xCELLigence system) proved that the influence of callus extract on the fibroblasts was dose-dependent. The evaluated level of fibroblasts proliferation rate after 72 h of incubation with callus extract at concentration 12.5 µg L^−1^ was the highest compared to all the analyzed ligands. Moreover, callus extract administrated for 72 h caused a significant increase in the proliferation rate in comparison with the control group (5.7 ± 0.1 vs. 4.4 ± 0.9; *p* < 0.01). The preliminary studies carried out may suggest that the callus extract rich in triterpenoids may be a potential source of cosmetic ingredients with a beneficial effect on human skin.

## 1. Introduction

*Chaenomeles japonica* (Thunb.) Lindl. ex Spach belongs to the subfamily Maloideae of the family Rosaceae. *C. japonica*, a dwarf shrub that naturally occurs in Central and South Japan [1,2]. In Europe, the plant was domesticated in the 19th century and has been appreciated for its ornamental value [3]. The high content of vitamin C, organic acids, phenolic compounds, dietary fiber, pectin, simple sugars of the fruits of *C. japonica*, make them well suited for industrial processing [4,5,6].

To date, the phytochemical studies of *C. japonica* have established the presence of bioactive compounds in selected raw materials from the wild plant—epicatechin, roseoside, monoterpene glucosides and leucoanthocyanin in fruits; epicatechin and flavonol glycosides in leaves; and daucosterol, three triterpenes—ursolic, oleanolic, pomolic acids, and epicatechin, prunasin in roots [7]. Among all representatives of the genus *Chaenomeles*, in the traditional medicine of the Far East, the fruits of *Chaenomeles speciosa* were used for centuries as ‘Mugua’, while the fruits of *C. japonica* have been used as an astringent and in stomach diseases [8]. Moreover, the extract from seeds of *C. japonica* is on the list of cosmetic ingredients approved for use in the European Union, acting as a supplement that nourishes the skin [9].

There is a growing interest in pentacyclic triterpenoids due to their interesting potential biological and pharmaceutical properties. Oleanolic acid and its isomer—ursolic acid—have long been known to be anti-inflammatory, hepato-protective, and anti-hyperlipidemic in the traditional medicine of Asia. Moreover, recently there have been many studies on the antiviral, antimicrobial and anticancer activities [10], and the anti-aging effect [11]. Betulinic acid was reported for its cosmetic properties [12]. The production of pentacyclic triterpenoids in vitro cultures (callus and cell suspension cultures) of various plants has been investigated. Several studies showed the production of oleanolic, ursolic and betulic acids by plants in vitro cultures [13].

Polyphenols are a group of common bioactive secondary metabolites widely distributed in plants. They exhibit a great diversity and are divided into several classes. The phenolic constituents have a wide range of biological activities, mainly attributed to their antioxidant potential [14]. Polyphenols possess a broad spectrum of activities applied in cosmetology—skin cell renewal, stimulation of collagen and elastin synthesis or elimination of oxidative stress. For these reasons, they have been perceived as effective anti-aging agents [15].

Therefore, callus and cell suspension cultures are widely applied in the investigation of the production of the high-value secondary metabolites, which may be used as cosmeceuticals, nutraceuticals, and pharmaceuticals. Plant cell cultures, with the continuous and reliable accumulation of desired bioactive compounds, are promising; an alternative to intact plants, sources for the production of the plant-derived drugs of industrial importance. In comparison with the conventional cultivation of plants, plant cell cultures offer an independent—of geographical and environmental factors—supply of uniform biomass with enhanced production of active constituents. Plant in vitro cultures ensure a rational utilization of biodiversity [16,17].

Fibroblasts, which were the object of this research, are the most common cells of connective tissue. The number of fibroblasts in the human dermis decreases with age and it is partially due to the reduced proliferation [18]. The research for finding natural substances with beneficial effects on the biological activities of skin fibroblasts is still needed.

The aim of this study was to investigate the effect of the well-characterized and rich in pentacyclic triterpenoids callus extract (line A2) of *Chaenomeles japonica* on the viability, morphology, and proliferation of normal human skin fibroblasts. For this reason, the authors initiated, established, and selected callus lines determined the main compounds in four lines of callus and evaluated the antioxidant activity of the extracts. The callus line A2 was chosen for the fibroblast-based experiments.

## 2. Results

**Figure 1:**
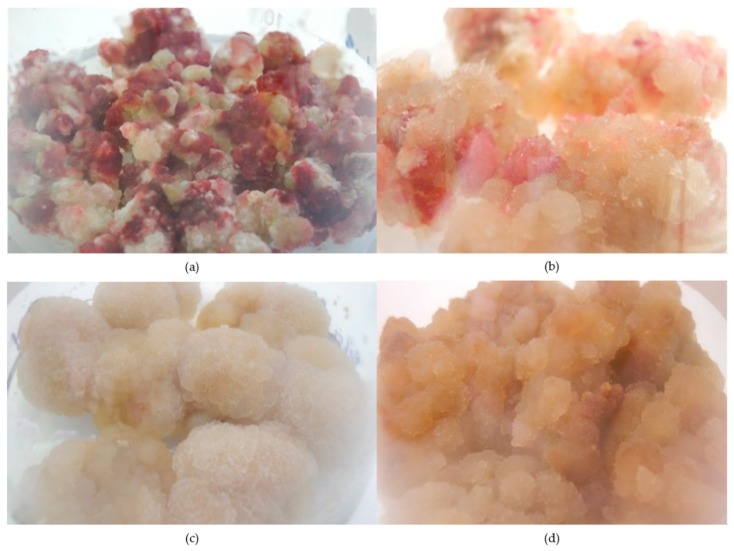
The callus lines of *Chaenomeles japonica* cultured in vitro on MS (Murashige and Skoog) media (**a**) Line A1: MS + 1.0 mg L^−1^ 2,4 D + 0.1 mg L^−1^ Kin (light); (**b**) Line A2: MS + 1.0 mg L^−1^ 2,4 D + 0.1 mg L^−1^ Kin (dark); (**c**) Line B: MS + 2.0 mg L^−1^ 2,4 D + 0.2 mg L^−1^ NAA (**d**) Line C: MS + 1.0 mg L^−1^ DIC.

The in vitro cultures of *C. japonica* were initiated from the seeds of intact plants (according to Reference [19]). The leaves from the shoot cultures (the stage of micro-propagation) were employed for the establishment of the callus. The induction of the callus occurred on all explants submitted to different plant growth regulators (100% efficiency). The callus biomass was friable and varied in color (Figure 1).

For subsequent experiments the leaf-derived callus was maintained on the three selected MS (Murashige and Skoog) media enriched with the phytohormones: MS + 1.0 mg L^−1^ 2,4D (2,4-dichlorophenoxyacetic acid) + 0.1 mg L^−1^ KIN (kinetin/ 6-furfurylaminoaminopurine) (kept in the light: A1 line, in the dark: A2 line), MS + 2.0 mg L^−1^ 2,4D + 0.2 mg L^−1^ NAA (α-naphthaleneacetic acid) (kept in the dark: B line), and MS + 1.0 mg L^−1^ DIC (dicamba/ 3,6-dichloro-2-methoxybenzoic acid) (kept in the dark: C line).

On these selected growing media, the biomass of the studied lines of callus intensively increased, reaching high rates of cell proliferation during the latest passages. The callus growth index depended on the line tested and ranged from 844.98 ± 28.62% to 893.90 ± 21.68% (Table 1, Figure 1).

**Figure 2:**
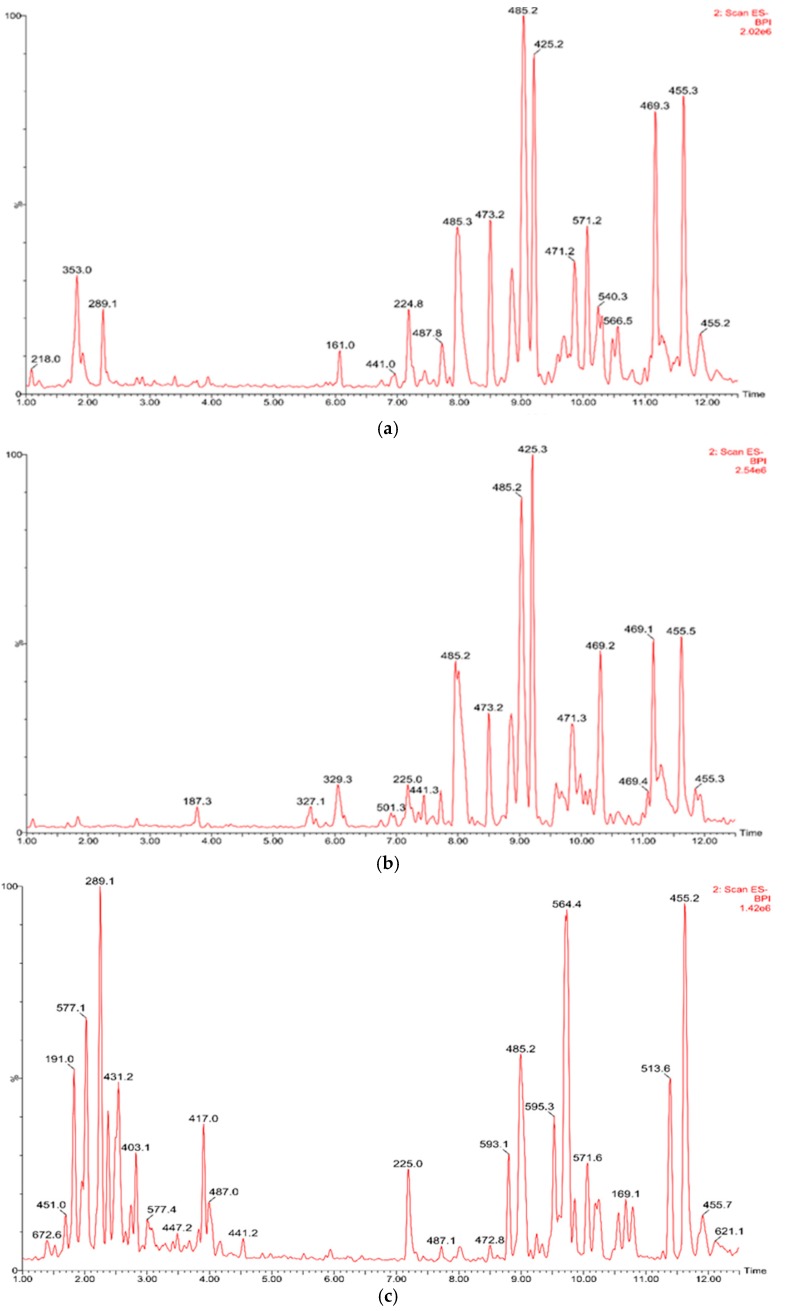
The High Performance Liquid Chromatography Tandem Mass Spectrometry (HPLC-MS/MS) chromatograms of extracts from *Chaenomeles japonica* (**a**) callus A1 (**b**) callus A2 (**c**) fruits.

Although the A2 line callus was characterized by the lowest concentration of polyphenols, mainly phenolic acids (the sum of chlorogenic acid, dicaffeoylquinic acid, hexoside quercetin and epicatechin was 0.21 mg g^−1^ *d.w*., the total content of phenolic acids was 1.67 ± 0.03 mg CA g^−1^) and a negligible level of flavonoids (the total content of flavonoids was 0.47 ± 0.01 mg QE g^−1^), and thus the lowest antioxidant activity (the CUPric Reducing Antioxidant Capacity assay—CUPRAC: IC_0.5_ 187.70 µg mL^−1^, the Ferric Reducing Antioxidant Power assay—FRAP: IC_0.5_ 168.08 µg mL^−1^, the1,1-diphenyl-1-picrylhydrazyl Radical Scavenging assay—DPPH: IC_50_ 516.88 µg mL^−1^). It, however, had the highest content of pentacyclic triterpenoids (the sum of ursolic acid, oleanolic acid and betulinic acid −9.81 ± 0.89 mg g^−1^ *d.w*.) (Table 2, Table 3, Table 4 and Table 5; Figure 2) and on this basis, was selected for further research of biological activities on fibroblasts.

**Figure 3:**
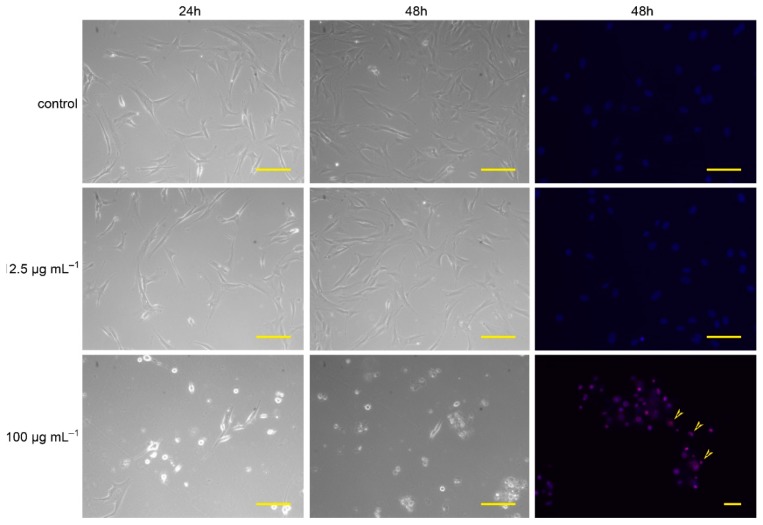
The human skin fibroblast cell morphology. The left column and the middle column show untreated control cells and cells stimulated with *C. japonica* callus extract at a final concentration of 12.5 and 100 µg mL^−1^ after 24 and 48 h of incubation, respectively. The right column shows cells stained with propidium iodide/Hoechst33342 after 48 h of incubation with *C. japonica* callus extract at a final concentration of 12.5 and 100 µg mL^−1^; yellow scale bar = 50 µm; yellow arrows indicate the dead cells stained with propidium iodide.

Figure 3 shows the morphology of the control fibroblasts and those treated with *C. japonica* extract from the callus A2 line in the selected concentrations and time intervals.

It can be seen that non-stimulated cells and fibroblasts treated with a callus extract at a lower concentration (12.5 μg mL^−1^) were characterized by a correct, elongated shape. The cells contained centrally located large and round nuclei that revealed chromatin with a granular structure. The morphology of the fibroblasts treated with 100 μg mL^−1^ extract was characterized by an incorrect morphology with narrow projections. Many dead cells were observed among these fibroblasts.

**Figure 4:**
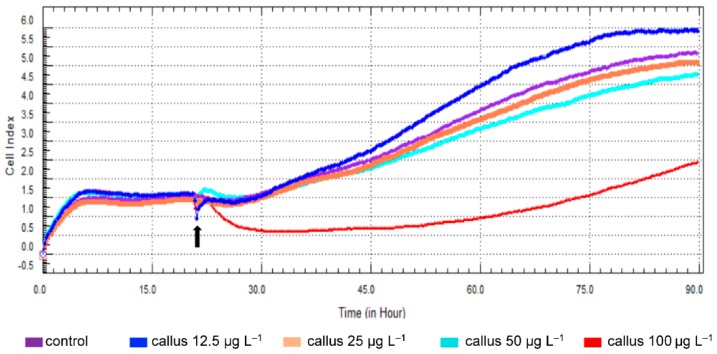
The real-time, label-free monitoring of the callus extracts effects of on CRTL-2522 human skin fibroblast proliferation using the xCELLigance system. A representative graph is shown. CRTL-2522 cells were treated with various concentrations of callus extract. The cell index value was monitored in real-time for 72 h (black arrow indicates the time of callus extract administration).

As a result of the microscopic observations of the fibroblast morphology (Figure 3) and the evaluation of the proliferation ratio (Figure 4), it can be noticed that the influence of *C. japonica* callus extract on the fibroblasts was dose-dependent.

Cell proliferation assays were performed using the xCELLigence system (ACEA Biosciences Inc., San Diego, CA, USA). After seeding CRL-2522 cells into the wells, the mean impedance change (*n* = 5) was measured. No significant differences in the proliferation rate among all analyzed groups were observed before stimulation (*p* > 0.05). Impedance was monitored every 15 min. Only four post stimulation points were analyzed: at 12, 24, 48, and 72 h to improve the clarity of the results presentation. The control cells showed a significant cell index increase, which, 12 h after stimulation, attained 1.4 ± 0.4 and increased to 4.4 ±1.2 after 72 h of incubation (*p* < 0.001).

RTCA DP software (Version 1.2.1, ACEA Biosciences Inc., San Diego, CA, USA) analysis performed after 12, 24, 48, or 72 h incubation showed a significant decrease of the cell index (CI) in case of cells stimulated with callus extract at final concentrations 100 or 50 µg L^−1^ in comparison to control. However, the rate of CI in CRL-2522 cells stimulated with callus extract of 12.5 µg L^−1^ was higher in comparison to the control cells in all time intervals (Table 6).

The representative graph, comparing the rate of CI in callus extracts stimulated CRL-2522 cells, was presented in Figure 4. The evaluated level of the fibroblast proliferation rate after 72 h of incubation with 12.5 µg L^−1^ callus extract was the highest compared to all analyzed ligands. Moreover, the callus extract administrated for 72 h caused a significant increase in the proliferation rate in comparison with the control group (5.7 ± 0.1 vs. 4.4 ± 0.9; *p* < 0.01).

Kinetin (N6-furfuryladenine), known from the literature as a small-molecule plant compound that acts as a growth factor, has been selected as a reference substance. It is one of the cytokinin compounds, where anti-aging effects on cultured human skin cells have been reported [20,21]. However, in our study after kinetin stimulation, no difference between the proliferation rate of CRL-2522 fibroblasts and the control cells was observed.

## 3. Discussion

The beneficial effect of the *C. japonica* callus extract on fibroblasts may be related to the compounds present in the biomass, in which pentacyclic triterpenoids and phenolic compounds predominate. The preliminary studies carried out may suggest that the callus extract rich in triterpenoids may be a potential source of cosmetic ingredients with a beneficial effect on human skin.

As it is known from the literature, ursolic and oleanolic acids (those compounds present in extracts from the callus and fruits of *C. japonica*), have demonstrated activity on human fibroblasts [22]. Oleanolic acid with less cytotoxicity than ursolic acid can be used as a complementary ingredient in products intended for dermal use [22]. However, the liposome-encapsulated ursolic acid increased the collagen synthesis of dermal fibroblasts and it is a promising agent with a wide variety of applications, for example, in anti-aging skin care products [23]. Moreover, the extract of *Manilkara bidentate*, with a beneficial effect on collagen and fibronectin synthesis and the extract of birch (*Betula* sp.) bark, both rich in pentacyclic triterpenes, have been considered as anti-aging ingredients for the cosmetic industry [24,25]. Asiaticoside, an active compound belonging to pentacyclic terpenoids from *Centella asiatica*, has enhanced the number of normal human dermal fibroblasts in the in vitro system [26]. The *Terminalia chebula* fruit extract is rich in phenolic acids and flavonol glycosides. Fibroblasts treated with extracts of this species at concentrations of 10–100 μg mL^−1^ survived and were divided even up to day 21 of culture. The cytoprotective effect of the extracts of this plant has been demonstrated in combination with high antioxidant activity. Treated fibroblasts were characterized by a normal morphology and good parameters of growth [27]. The extract from *Symphytum officinale* rich in phenolic acids (including hydroxybenzoic acid, chlorogenic acid, and p-coumaric acid) characterized by the lack of cytotoxicity, stimulates the viability and metabolism of human skin fibroblast cells [28]. Furthermore, the extract of *Persea americana* seeds, mostly composed of quinic acid, chlorogenic acid, and their isomers, enhanced the proliferation rate of human skin fibroblasts and keratinocytes and did not affect their cytotoxicity [29]. The extracts from *C. japonica* are rich in chlorogenic acid, which is known from the literature as a compound affecting, among others, the proliferation of fibroblasts and preferably, acting to improve skin regeneration [30].

The authors believe that the *C. japonica* extracts are worthy to be investigated in regard to their properties to positively affect the skin, which is related to the presence of valuable compounds. Many researchers suggest that extracts rich in bioactive compounds, as opposed to single compounds, can be characterized by synergistic effects on cells, increasing their proliferation rate and scavenging free radicals [27]. A breakthrough in understanding human skin cells (e.g., fibroblasts, keratinocytes) treated with plant-derived extracts with well-defined and natural ingredients offers a pathway for anti-aging product development. Our research brings us closer to understanding how the *C. japonica* callus extract may influence the cultured fibroblasts, cells of connective tissue, which number decreases during the aging process of the skin.

## 4. Materials and Methods

### 4.1. Plant Material, Surface Disinfection, and Culture Media Establishment

The voucher specimens of *C. japonica* (no. 1526/2016) are deposited in the Herbarium of the Medicinal Plant Garden in the Institute of Natural Fibers and Medicinal Plants in Poznan, Poland. The mature fruits of *C. japonica* were collected from the old shrub growing in the garden (52°21′55.4″ N 17°00′12.5″ E; 52.365381, 17.003471) in Poznan (Poland), in 2013. For the aseptic culture initiation, the seeds were isolated from fresh fruits, surface disinfected, and used as primary explants. The isolated seeds were washed in distilled water for 5 min followed by submerging in 70% (*v*/*v*) ethanol for 30 s to degrease the seeds and placed on lignin in a thermostat (26 °C) for 24 h to swell the seeds. After this pretreatment, the seeds were disinfected with commercial bleach at a 50% concentration for 20 min with two drops of Tween 20. They were finally rinsed 5 times in sterilized bi-distilled water and transferred to Murashige and Skoog (MS) [31] medium to obtain the aseptic seedlings, whose parts were the secondary explants for in vitro cultures establishment. All types of culture media consisted of MS basal media solidified with 0.8% (*w*/*v*) agar (Sigma-Aldrich, Saint. Louis, MO, USA) supplemented with 30 g L^−1^ (*w*/*v*) sucrose and plant growth regulators at various concentrations. All plant growth regulators originated from Sigma-Aldrich (St. Louis, Saint Louis, MO, USA). After adjusting pH to 5.8, the media were autoclaved at 121 °C for 20 min at 105 kPa. Cultures were incubated in a growth chamber (16/8 h photoperiod, 55 μmol m^−2^ s^−1^ light, temp. 21 ± 2 °C).

### 4.2. Induction and Maintenance of Callus

The callus culture was initiated from the leaves of the micropropagated plantlet explants on various solid MS media (1.0 mg L^−1^ 2,4-D + 0.1 mg L^−1^ KIN, 1.0 mg L^−1^ 2,4-D + 1.0 mg L^−1^ KIN, 2.0 mg L^−1^ 2,4-D + 1.0 mg L^−1^ KIN, 0.5 mg L^−1^ 2,4-D + 0.05 mg L^−1^ NAA, 1.0 mg L^−1^ 2,4-D + 0.1 mg L^−1^ NAA, 2.0 mg L^−1^ 2,4-D + 0.2 mg L^−1^ NAA, 1.0 mg L^−1^ 2,4-D, 2.0 mg L^−1^ 2,4-D, 1.0 mg L^−1^ DIC, or 2.0 mg L^−1^ DIC) and then established on selected MS with 1.0 mg L^−1^ 2,4D + 0.1 mg L^−1^ KIN (light condition) (line A1), MS with 1.0 mg L^−1^ 2,4D + 0.1 mg L^−1^ KIN (dark condition) (line A2), MS with 2.0 mg L^−1^ 2,4D + 0.2 mg L^−1^ NAA (dark condition) (line B), MS + 1.0 mg L^−1^ DIC (dark condition) (line C). Sub-cultures were performed at 4-week intervals. Callus initiation and its development were first observed visually and then when callus culture became stabilized (23–25 passages), the growth callus index was calculated by the following equation: Growth index = (final dry cell weight − initial dry cell weight)/initial dry cell weight.

The stable and homogenous leaf-derived callus (line A2) growing in the darkness, on a medium MS with 1.0 mg L^−1^ 2,4D + 0.1 mg L^−1^ KIN (26^th^ subculture), after phytochemical analyses were chosen for the biological experiments.

### 4.3. Phytochemical Analysis

Lyophilized plant samples (100 mg) were ground in a mortar and extracted with 80% (*v*/*v*) methanol, using the accelerated solvent extraction system (ASE 200, Dionex, Sunnyvale, CA, USA). Extraction was carried out at 100 °C, operating pressure was 1500 psi. The extracts were evaporated to dryness, suspended in 5% MeOH, and subjected to solid phase extraction (SPE) on Waters SepPak Classic cartridges equilibrated with 5% MeOH. The analytes were eluted with 95% MeOH, evaporated to dryness and reconstituted in 3.000 mL of 90% MeOH. The samples were then stored in −20 °C and centrifuged at 23,000 x g for 15 min before analysis. UHPLC-DAD-ESI-MS analyzes were performed using an ACQUITY UPLC^®^ chromatographic system (Waters Corp., Milford, MA, USA), equipped with a triple quadrupole mass detector.

For the determination of the content of oleanolic, ursolic, and betulinic acids, an ACQUITY HSS C18 column (2.1 × 100 mm, 100 Å, 1.8 µm; Waters, Milford, MA, USA) was used, the flow rate was 0.400 mL min^−1^ (30 °C), and the injection volume was 2.5 µL. Analytes were separated isocratically for 11.9 min using 80% methanol containing 0.1% formic acid. The column was subsequently washed with 99% MeOH (0.1% formic acid) (12−13 min) and the initial conditions were restored (13.1–15.0 min). Triterpenoid acids were detected by MS in the negative ionization mode, using the Selected Ion Monitoring (SIM) method. The following MS settings were applied: capillary voltage was 2.8 kV; cone voltage was 80 V; source temperature was 140 °C, desolvation temperature was 350 °C, cone gas flow (nitrogen) was 100 L h^−1^, desolvation gas flow was 800 L h^−1^. The content of ursolic and oleanolic acid in the investigated samples was determined by external calibration (oleanolic acid: y = −456,02 x 2 + 52103x + 70463, R2 = 0,991; ursolic acid: y = −339,1x2 + 40079x + 67659, R2 = 0,990). The betulinic acid content was expressed as oleanolic acid equivalent. Phenolic compounds were determined using an ACQUITY BEH C18 column (2.1 × 100 mm, 130 Å, 1.7 µm; Waters), maintained at 50 °C. The injection volume was 2.5 µL. Gradient elution was applied using 0.1% formic acid in Milli-Q water as solvent A and acetonitrile with 0.1% formic acid as solvent B, according to the following program: 0–0.5 min, 7% B; 0.5–11.9 min, 7–80% B; 11.9–12.0 min, 80–95% B; 12.0–13.0, 95% B; 13.0–13.1 min., 95–7% B; 13.1–15 min., 7% B.

The MS analysis was carried out using the scanning method in the positive and negative ionization mode. The MS settings for the negative ionization mode were as follows: the capillary voltage was 2.8 kV; cone voltage was 45 V; source temperature was 140 °C, desolvation temperature was 350 °C, cone gas flow (nitrogen) was 100 L h^−1^, desolvation gas flow was 800 L h^−1^; positive ionization: capillary voltage was 3.1 kV, cone voltage was 60 V, other settings were as above. Constituents of the extracts were tentatively identified on the basis of their UV spectra and/or MS data. Chlorogenic acid was quantified on the basis of UV chromatograms, using a calibration curve of chlorogenic acid (y = 182.94x – 560.94, R2 = 0.9981). The contents of other phenolics were expressed as chlorogenic acid equivalents.

Methanol (isocratic and gradient grade), acetonitrile (LC-MS grade), formic acid (LC-MS grade) were from Merck Millipore (Darmstadt, Germany). Standards of oleanolic, ursolic, and chlorogenic acid were from Sigma-Aldrich (Saint Louis, MO, USA).

Extractions and analyzes were performed in triplicate, while the presented results are means-based with standard deviation.

### 4.4. Determination of Total Polyphenolic, Phenolic Acid, and Flavonoid Contents

Samples of lyophilized and powdered biomass were extracted three times with a matched quantity of 80% (*v*/*v*) ethanol for 60 min at the boiling point temperature of the extractive mixture under reflux. The extracts were evaporated to dryness under a reduced pressure.

The content of phenolics in the ethanol-water extracts (80%, *v*/*v*) was determined spectrophotometrically using Dóka and Bicanic’s (2002) [32] modified method, with the Folin-Ciocalteu reagent. Briefly, 0.05 mL of the tested extract was mixed with 3.7 mL of distilled water and 0.25 mL of the Folin-Ciocalteu reagent. The mixture was shaken vigorously and a 20% (*w*/*v*) sodium dicarbonate solution (1.0 mL) was added after 1 min. Next, the samples were incubated in the dark for 30 min at room temperature. The absorbance was measured at 760 nm. The concentration of the total phenolic compounds in the extract was expressed as mg of the gallic acid equivalent per gram of the dry plant material weight from a calibration curve of gallic acid (y = 9.8399x + 0.0289, R2 = 9993) in a concentration rate (20–80 µg 10 mL^−1^). The values are expressed as the mean of 6 replications ± SD.

The content of phenolic acids in the ethanol-water extracts (80%, *v*/*v*) was determined by a spectrophotometric method described in Polish Pharmacopeia VI (2002) [33] with Arnov’s reagent (10.0 g of sodium molybdate, 10.0 g of sodium nitrite in 100.0 mL of water). The sample (1.0 mL) was pipetted into a 10.0 mL volumetric flask containing 5.0 mL water; next, 1.0 mL HCl (18 g L^−1^), 1.0 mL Arnov’s reagent and 1.0 mL NaOH (40 g L^−1^) were added. The volume was made up to 10.0 mL with distilled water. The absorbance was measured at 490 nm. The total concentration of the phenolic acid content was expressed as mg of caffeic acid (40–200 µg 10 mL^−1^) per gram of dry plant material weight from a calibration curve of caffeic acid (y = 11.382x + 0.0746, R2 = 0.9834) in a concentration rate of 40–300 µg 10 mL^−1^. The values are expressed as the mean of 5 replications ± SD.

The content of flavonoids in the ethanol-water extracts (80%, *v*/*v*) was determined as described by Meda et al. (2005) [34]. Briefly, equal volumes of 2% AlCl_3_ in methanol (0.7 mL) and the extract (0.7 mL) were mixed and left for 10 min. The absorbance was measured at 415 nm using a blank sample of water and methanol without AlCl_3_. The total concentration of the flavonoid content was expressed as mg of quercetin per gram of the dry plant material weight, from a calibration curve of quercetin (y = 0.046x − 0.0199, R2 = 0.9991) in a concentration range (3.125–25 µg mL^−1^). The values are expressed as the mean of 6 replications ± SD.

### 4.5. Determination of Antioxidant Activity DPPH, FRAP, CUPRAC Assays

The DPPH assay was conducted according to Studzińska-Sroka et al. [35] with modifications. Briefly, 25 μL of the dry extracts dissolved in DMSO (Sigma-Aldrich, Saint-Louis, MO, USA) at different concentrations (0.3125–5 mg/mL) were mixed with a 175 μL of DPPH (Sigma-Aldrich, St. Louis, MO, USA) solution (39 mg 50 mL^−1^ of MeOH; the final assay concentrations were 78.13–625 μg mL^−1^). The reaction mixture was shaken and incubated in the dark, at room temperature for 30 min. Absorbance was measured at 517 nm against the blank (25 μL of DMSO and 175 μL of MeOH). The control contains 25 μL of DMSO and 175 μL of DPPH solution. The inhibition of the DPPH radical by the sample was calculated according to the following formula: DPPH scavenging activity (%) = (A0−A1)/A0 × 100%, where A0 is the absorbance of the control and A1 is the absorbance of the sample. Analyses were performed in six replicates. Vitamin C was used as a standard (7.5–80 μg mL^−1^; the final assay concentrations were 0.9375–15 μg mL^−1^). The results were expressed as the IC_50_ value corresponds to the concentration of the extract required to inhibit DPPH radical formation by 50% and was determined from the linear regression analysis.

The FRAP assay was performed according to Tiveron et al. [36] with some modifications. The stock solutions of FRAP reagent included 300 mM acetate buffer (pH 3.6), 10 mM 2,4,6-Tris(2-pyridyl)-s-triazine (TPTZ, Sigma-Aldrich, Saint-Louis, MO, USA) solution in 40 mM HCl, and 20 mM FeCl_3_·6H_2_O (Sigma-Aldrich, Saint-Louis, MO, USA) solution. The working FRAP solution was freshly prepared by mixing 25 mL of acetate buffer, 2.5 mL of TPTZ solution, and 2.5 mL of FeCl_3_·6H_2_O solution, and then warmed at 37 °C before usage. Briefly, 25 μL of the tested extracts were dissolved in DMSO at different concentrations (0.0125–3.2 mg of extract/mL) was mixed with 175 μL of the FRAP solution (the final assay concentrations were 3.125–400 μg mL^−1^), shaken and incubated at 37 °C for 30 min in the dark condition. Then the absorbance was read at 593 nm. The analysis was performed in six replicates. Vitamin C was used as a standard (0.0125-0.2 mg mL^−1^; the final assay concentrations were 0.195–3.125 μg mL^−1^). The results were expressed as the IC_0.5_, which corresponds to the extract concentration required to produce 0.5 O.D. value.

The CUPRAC assay was conducted according to Apak et al. [37] with modifications. The stock solutions of the CUPRAC reagent included equal parts of acetate buffer (pH = 7.0), 7.5 mM neocuproine (Sigma-Aldrich, St. Louis, MO, USA) solution in 96% ethanol, and 10 mM CuCl_2_·H_2_0 (Avantor Performance Materials, Gliwice, Poland) solution. Briefly, 50 μL of the dry tested extracts dissolved in DMSO at different concentrations (0.0125–1.6 mg mL^−1^), were mixed with 150 μL of CUPRAC solution (the final assay concentrations were 3.125–400 μg mL^−1^), shaken and incubated at room temperature for 30 min in the dark condition. Then the absorbance was read at 450 nm. The analysis was performed in six replicates. Vitamin C was used as a standard (0.0125–0.1 mg mL^−1^; the final assay concentrations were 3.125–25 μg mL^−1^). The results were expressed as the IC_0.5_ which corresponds to the extract concentration required to produce 0.5 O.D. value.

### 4.6. Biological Activity on Fibroblasts

The research was carried out on human skin fibroblasts established from male newborns normal foreskin (CRL-2522, ATCC, Manassas, VA, USA). Fibroblasts were cultured in EMEM medium (Corning, New York, NY, USA) supplemented with 100 μg mL^−1^ streptomycin (Cytogen, Sinn, Germany), 100 μg mL^−1^ penicillin (Cytogen, Sinn, Germany), 2 mmol L^−1^ L-glutamine (Cytogen, Sinn, Germany), and 10% fetal bovine serum (FBS, Corning, NY, USA). The cell line was maintained in aseptic conditions at 37 °C in a humidified, 5% CO_2_ incubator and confirmed free of mycoplasma contamination through regular testing (Mycoplasma PCR Test Kit, AppliChem, Darmstadt, Germany). Cells were cultured until 90% confluence. At this point, they were detached from culture dishes with a trypsin solution (2.5%, Biowest, Riverside, MO, USA). After 3 min of incubation, the trypsin was removed, complete growth medium was added, and the cell suspension was transferred into sterile Petri dishes. Cells prepared due to this procedure were used for further investigations.

The cell proliferation ratio was evaluated using the xCELLigence RTCA DP system (Roche Diagnostics, Mannheim, Germany; ACEA Biosciences, San Diego, CA, USA). Real-time measurements of cell proliferation were conducted using 16-well plates (F. Hoffmann-La Roche Ltd., Basel, Switzerland; ACEA Biosciences, San Diego, CA 92121 USA). Gold microelectrodes are attached at the bottom of each well for the impedance-based detection of cells biological status, including attachment and cell number. The voltage of about 20 mV applied to the electrodes during the test does not affect the examined cells. The electrical impedance was measured by the integrated software of the xCELLigence RTCA DP system as a cell index (CI). Cells were seeded into the wells at a density of 10,000 cells/well.

Afterward, the cells were treated with callus extracts/fruit extracts/kinetin at different final concentrations: 100, 50, 25, 12.5 μg mL^−1^. The control consisted of cells cultured in a medium without addition of the above ligands. Cell proliferation was monitored every 15 min for 72 h. Cells morphology and viability were evaluated using an Axio Vert.A1 microscope (10× and 20× magnitude, Zeiss, Jena, Germany) by means of propidium iodide/Hoechst33342 double staining after 48 h of incubation with different ligand concentrations: 100; 12.5 μg mL^−1^ and subsequently analyzed with AxioVision software (Zeiss, Jena, Germany). Microscopy analysis was performed on the same selected area on the Petri dish before and after propidium iodide/Hoechst33342 staining.

### 4.7. Statistical Analysis

The collected data from the biotechnological experiments were subjected to a one-way analysis of variance ANOVA, followed by Duncan’s post-hoc test. A two-sided *P*-value of 0.05 was used to declare statistical significance. The Shapiro–Wilk test was used as the normality test of continuous variables. Homogeneity of variance was assessed with the Levene test. A one-way ANOVA followed by Tukey (RIR) post hoc test was used to analyze the relationship between various ligands. Repeated measures of one-way ANOVA with the Tukey–Kramer multiple comparisons test were used to evaluate changes in time for particular ligands. The analysis was made by using Statistica 10 software (StatSoft Inc., Tulsa, OK, USA). Data were presented as mean ± standard deviation and considered statistically significant at *p* < 0.05.

## 5. Conclusions

The stimulation of the proliferation of human skin fibroblasts by *C. japonica* callus extracts may indicate the prospects for their use in the design of new cosmetic preparations based on plant raw materials.

## Figures and Tables

**Table 1:** The growth parameters of selected lines of *Chaenomeles japonica* callus growing on various variants of MS (Murashige and Skoog) medium.

Callus Line	Callus Growth Index (%) ± Standard Error			
	Passage 23	Passage 24	Passage 25	Mean
A1	859.93 ± 13.82	842.10 ± 29.31	970.23 ± 37.66	893.90 ^ns^ ± 21.68
A2	863.81 ± 44.37	841.22 ± 65.35	849.62 ± 35.53	849.62 ± 35.53
B	689.00 ± 55.30	915.40 ± 49.58	863.02 ± 38.56	863.02 ± 38.56
C	849.46 ± 47.22	841.63 ± 31.74	844.98 ± 28.62	844.98 ± 28.62

Ns: no significant differences at P = 0.05 (Duncan’s test).

**Table 2:** The content (mg g^−1^ *d.w*.) of selected polyphenols in four lines of *Chaenomeles japonica* callus.

Callus Line/Plant Material	Polyphenols [Content mg g^−1^ ± Standard Error]				
	CA	DCCA	HQ	EC	Sum of CA, DCCA, HQ, EC
A1	1.09 ± 0.08	0.08 ± 0.01	0.06 ± 0.01	0.07 ± 0.01	1.30 ± 0.10
A2	0.12 ± 0.01	0.05 ± 0.01	0.05 ± 0.00	trace	0.21 ± 0.02
B	2.68 ± 0.15	-	0.06 ± 0.01	0.08 ± 0.02	2.81 ± 0.16
C	0.93 ± 0.04	0.25 ± 0.01	0.10 ± 0.00	0.09 ± 0.01	1.36 ± 0.05
Fruit	0.96 ± 0.05	-	-	0.30 ± 0.06	1.25 ± 0.09

CA: chlorogenic acid, DCCA: dicaffeoylquinic acid, HQ: hexoside quercetin, EC: epicatechin.

**Table 3:** The content (mg g^−1^ *d.w*.) of selected pentacyclic triterpenes in four lines of *Chaenomeles japonica* callus.

Callus Line/Plant Material	Pentacyclic Triterpenes [Content mg g^−1^ ± Standard Error]			
	UA	OA	BA	Sum of UA, OA, BA
A1	4.27 ± 0.65	3.92 ± 0.37	0.43 ± 0.03	8.62 ± 1.15
A2	5.00 ± 0.61	4.35 ± 0.30	0.46 ± 0.03	9.81 ± 0.89
B	1.22 ± 0.09	1.26 ± 0.09	0.22 ± 0.01	2.70 ± 0.17
C	2.27 ± 0.19	1.48 ± 0.11	0.25 ± 0.02	4.00 ± 0.29
Fruit	1.85 ± 0.19	1.51 ± 0.17	0.58 ± 0.05	3.94 ± 0.43

UA: ursolic acid, OA: oleanolic acid, BA: betulinic acid.

**Table 4:** The total content of total polyphenolics, phenolic acids, and flavonoids in four lines of *Chaenomeles japonica* callus.

Total	Content of Selected Groups of Compounds in Extracts [± Standard Error]				
	A1	A2	B	C	Fruit
POLYPHENOLS [mg GAE g^−1^]	20.60 ± 0.20	8.87 ± 0.21	41.05 ± 0.84	6.61 ± 0.57	52.50 ± 1.58
PHENOLIC ACIDS [mg CAE g^−1^]	22.48 ± 0.82	1.67 ± 0.03	7.11 ± 0.15	4.80 ± 0.07	18.02 ± 0.36
FLAVONOIDS [mg QE g^−1^]	0.60 ± 0.07	0.47 ± 0.01	0.986 ± 0.03	0.64 ± 0.02	0.33 ± 0.01

GAE: gallic acid equivalent, CAE: caffeic acid equivalent, QE: quercetin equivalent.

**Table 5:** The antioxidant effect of four lines of *Chaenomeles japonica* callus.

Plant Material	CUPRAC IC_0.5_ (μg mL^−1^)	FRAP IC_0.5_ (μg mL^−1^)	DPPH IC_50_ (μg mL^−1^)
A1	90.05	62.69	178.51
A2	187.70	168.08	516.88
B	117.18	92.72	305.49
C	110.03	75.14	234.00
Fruit	40.91	27.62	75.11
Vitamin C	9.28	5.03	5.48

**Table 6:** The effect of callus extracts of *Chaenomeles japonica*, kinetin, and fruit extracts on the proliferation rate of CRL-2522 cells.

Plant Material/Reference Compound	Cell Index Value Mean ± Standard Deviation			
	Incubation Time			
	12 h	24 h	48 h	72 h
**Control**	1.4 ± 0.4	1.9 ± 0.5	3.3 ± 0.5	4.4 ± 0.6
**Callus**				
(100 µg L^−1^)	0.2 ± 0.0 **	0.2 ± 0.0 **	0.2 ± 0.0 **	0.1 ± 0.1 **
(50 µg L^−1^)	0.5 ± 0.1 **	0.5 ± 0.1 **	0.8 ± 0.4 **	1.3 ± 0.9 **
(25 µg L^−1^)	1.4 ± 0.4	1.6 ± 0.6	2.9 ± 0.7	4.2 ± 0.4
(12.5 µg L^−1^)	1.7 ± 0.4	2.1 ± 0.5	3.8 ± 0.6	5.7 ± 0.1 *
**Kinetin**				
(100 µg L^−1^)	1.4 ± 0.0	1.4 ± 0.0	1.7 ± 0.1	1.5 ± 0.3
(50 µg L^−1^)	1.3 ± 0.3	1.6 ± 0.6	2.4 ± 1.0	3.1 ± 1.5
(25 µg L^−1^)	1.2 ± 0.6	1.3 ± 0.6	2.3 ± 1.1	3.5 ± 1.5
(12.5 µg L^−1^)	1.4 ± 0.6	1.5 ± 0.6	2.9 ± 1.2	4.3 ± 1.5
**Fruit**				
(100 µg L^−1^)	0.5 ± 0.1 **	0.5 ± 0.1 **	0.8 ± 0.5 **	0.8 ± 0.6 **
(50 µg L^−1^)	1.2 ± 0.8	1.3 ± 0.8	2.1 ± 1.3	2.6 ± 1.7
(25 µg L^−1^)	1.4 ± 0.5	1.5 ± 0.5	2.8 ± 0.5	4.3 ± 0.5
(12.5 µg L^−1^)	1.4 ± 0.6	1.6 ± 0.7	2.9 ± 1.0	4.3 ± 1.0
*P*-value	<0.0001	<0.0001	<0.0001	<0.0001

Cell index value was monitored using the xCELLigance system. Results are from five repeats. *P*-value calculated from one-way analysis of the variance; * *p* < 0.01 vs. control; ** *p* < 0.001 vs. control.
